# “If you can’t treat HPV, why test for it?” Women’s attitudes to the changing face of cervical cancer prevention: a focus group study

**DOI:** 10.1186/1472-6874-14-64

**Published:** 2014-05-06

**Authors:** Judith McRae, Cara Martin, John O’Leary, Linda Sharp

**Affiliations:** 1 National Cancer Registry, Building 6800, Cork Airport Business Park, Kinsale Road, Cork, Ireland; 2 Trinity College Dublin, Dublin, Ireland; 3 Coombe Women and Infants University Hospital, Dolphin’s Barn, Dublin 8, Ireland

**Keywords:** Cervical screening, HPV testing, Qualitative

## Abstract

**Background:**

The relationship between infection with high-risk strains of human papillomavirus (HPV) and cervical cancer is transforming prevention through HPV vaccination and HPV oncogenic testing. In Ireland, a national cervical cancer screening programme and HPV vaccination were recently launched; HPV testing is currently being integrated into the screening programme. Women’s views on the transformation of cervical cancer prevention have been relatively little investigated.

**Methods:**

Using qualitative focus groups, we determined women’s knowledge, attitudes towards, and acceptability of cervical cancer screening, HPV oncogenic testing and vaccination of HPV. Fifty nine women, recruited through primary care in Ireland, participated in ten focus groups. A dynamic topic guide was developed from literature reviewed. Women were provided with standardised information about HPV infection, HPV testing. Discussion transcripts were analysed thematically.

**Results:**

The primary themes that emerged regarding HPV infection were: knowledge, emotional response and societal influences; especially those of healthcare practitioners. Knowledge, logistics, and psychological impact were the primary themes relating to HPV testing. Women’s attitudes towards HPV testing changed during discussion as issues were explored, thus demonstrating the complexity of this issue; lack of existing treatment for HPV infection influenced women’s attitudes, attachment to existing cervical cancer screening also was a significant factor.

**Conclusions:**

Women currently have a strong attachment to cytology and any changes towards HPV primary testing will need to be managed carefully. To ensure that future cervical cancer prevention strategies will be acceptable to women, sufficient thought will have to be given to information provision and education. We identified the importance to women of healthcare practitioners’ opinions regarding HPV. Appropriate and timely information on HPV will be crucial in order to minimise possible psychological effects women may have.

## Background

Worldwide 530,000 new cervical cancers are diagnosed annually and there are 275,000 deaths from the disease [1]. The disease is largely preventable. Until recently the cornerstone of prevention was screening, using cervical cytology tests. However, the recent advent of the human papillomavirus (HPV) test and vaccination is transforming prevention strategies.

Genital HPV is a common sexually transmitted virus. Some strains cause genital warts and others, about 15 “high-risk” types; cause abnormal cervical cells that may eventually progress to cervical cancer. No specific treatment is available and most infections clear themselves [2].

Co-testing (i.e. primary screening using HPV and cytology tests) is now routinely recommended in USA [2]. Other countries are introducing HPV testing in triage of women with low-grade abnormal cytology (England [3]) and/or in follow-up of women treated for high-grade abnormal cytology (Scotland [4], Ireland [5,6], and others).

Two HPV prophylactic vaccines are currently licensed: Gardasil (Sanofi Pasteur MSD), and Cervarix (GSK Biologicals). Both vaccines target the most prevalent high-risk HPV strains, 16 and 18. Gardasil also targets HPV types 6 and 11 (which are linked with genital warts). The vaccines are given as a course of three injections over six-months [7]. While it is expected that these vaccines can prevent around 70% of cervical cancers, it is also recognised that widespread vaccination will not eliminate the need for some form of screening [8]. Vaccination programmes are in place in various countries [8], including Ireland [9].

Organised cytology-based screening has been effective in reducing incidence of and mortality from cervical cancer at the population-level [10,11]. That success, and the success of future prevention strategies, is predicated on achieving high levels of uptake among the target population. High uptake is dependent on women finding the strategies acceptable, but the effect of incorporating HPV testing into established screening programmes remains uncertain. Moreover, concerns have been expressed regarding the potential impact of HPV vaccination on future screening participation [8].

Women’s views on the transformation of cervical cancer prevention have been relatively little investigated. In order to inform policy makers and those tasked with service delivery, we aimed to explore women’s attitudes, knowledge and practices with regard to cervical cancer screening, HPV testing and vaccination, in the face of such changes. This study was the first in Ireland to examine women’s opinions on all aspects of cervical cancer prevention, and in particular on all aspects of HPV testing; HPV testing is in the process of being introduced into the national cervical cancer screening programme [9].

## Methods

The study setting was Ireland, which has a mixed public-private healthcare system. Organised cervical cancer screening commenced in the mid-western area in 2000, and the national programme, CervicalCheck, was rolled out in September 2008 providing free cytology tests to women aged 25 to 60 [5]. Prior to this, opportunistic screening was widespread.

Qualitative focus groups were used to permit in-depth exploration of women’s views and because interactions between group members may stimulate emergence of additional issues. Focus groups were conducted in urban, mixed and rural areas, with different socio-economic characteristics, during August 2007– August 2008. Women were recruited through general practices, primary care centres and well women centres using passive (posters in clinics) and active advertising (all women attendees during certain periods were given flyers by the clinic). Participation was open to women aged >17; previous experience of cervical cancer screening/cytology tests was not required. In order to ensure maximum diversity, women of a range of ages, and public and private patients, were recruited. Groups were organised until conceptual saturation was reached.

Women interested in participating returned their details by post to the research team who telephoned them to gather preliminary socio-demographic information and arrange a suitable date for the group. Women were not offered any financial incentive to participate or payment, but were offered (after the group) reimbursement for travel or child-minding costs if required.

Groups were held at locally convenient locations (e.g. general practices, hotels, civic centres). Women completed a consent form at the outset and anonymity and confidentiality were discussed. Groups lasted 90–150 minutes. A trained facilitator (JM) introduced discussion topics from the topic guide and a co-facilitator noted group dynamics and non-verbal communications. Each group discussed cervical cytology tests and cervical cancer screening, HPV infection, and then either HPV testing or HPV vaccination, with this topic chosen at random by the facilitator before the group started.

The topic guide was developed from review of literature on women’s attitudes, knowledge and awareness of cervical cancer screening and HPV. While the topic guide formed the basis of discussions, it was used dynamically, and allowed to evolve such that discussions in one group informed the topic guide for the next group. This helped to ensure that sufficient depth was reached.

Following discussion on cervical cytology tests and cervical cancer screening, the facilitator asked whether group members were aware of HPV and, if so, what they knew. Groups were then provided with a brief HPV information sheet (Additional file 1), which was also read aloud by the facilitator. The group then discussed HPV; and awareness of the link between sexual activity and HPV infection was explored explicitly using prompts. In the discussion on HPV testing, groups were invited to discuss: what is involved in testing, advantages and disadvantages, impact on screening, and psychological impact. Women were presented with three scenarios for discussion, relating to different potential uses of HPV testing: (a) as a primary test, (b) for women with mildly abnormal cytology to help decide if follow-up is needed, and (c) in women treated for abnormal cytology to help decide if further treatments or follow-up are required.

At the conclusion of the group each woman received a €20 shopping voucher to thank them for their time and participation; women had no prior knowledge of this. Women were provided with an information pack relating to the discussion topics in order to address any questions or alleviate any concerns, and were advised to contact their family doctor if they had any specific health concerns.

Discussions were audio-recorded, transcribed verbatim and anonymised. The analysis used a thematic approach [12,13] and was on-going and iterative, such that analysis of early focus groups informed the content of later groups to ensure sufficient depth was reached. To help ensure validity of coding categories and provide analytical rigour, two experienced qualitative researchers (including JM) independently reviewed the first two focus group transcripts, coded these and, in discussion, agreed the principal themes. The codes were applied to the rest of the dataset (by JM), but the code lists were also refined and developed as analysis progressed. Descriptive accounts of each group were prepared and the methods of Knodel [14] were used to identify more specific themes. Each theme was considered in the context of all of the groups.

Ethical approval was granted by the Irish College of General Practitioners. This study conforms to the RATS guidelines for qualitative research.

## Results

### Participants

Fifty nine women participated in ten focus groups (Additional file 2). Participants were aged from 17 to 69 years (mean = 42), two-thirds were married or cohabiting and education levels varied. Six women (10%) had never had a cervical cytology test. 31% were part of the public healthcare system.

### HPV infection

A few women had heard of HPV infection prior to the group, generally in relation to colposcopy or HPV vaccination, but most had not. Even those who had heard of HPV had unanswered questions about issues such as the source of infection, transmission and re-infection. The different HPV strains and risk factors were unknown by nearly all women. In general women were more concerned about cytological test results and cervical cancer than HPV infection.

Three primary themes relating to HPV infection emerged: *knowledge, emotional responses, societal influences* (Additional file 3).

### Knowledge

Women often wondered why they had not been told about or heard of HPV. They were eager to obtain more information, but were wary that inadequately explained information could result in negative psychological effects (e.g. worries, fear). Women expressed conflicting opinions about informing the population at large about the link between HPV and cervical cancer: some were in favour of providing comprehensive information and others were more cautious because this may cause fear. Some women thought if the relationship between HPV infection and cervical cancer was given greater prominence, it might encourage women to attend for cytology tests. Women expressed a feeling of security because of the high prevalence of HPV. This prevalence made women reluctant to consider, or label, infection with HPV a sexually transmitted disease (STD).

### Emotional responses

Women often expressed shock on learning of the prevalence of HPV infection. They felt their emotional response to HPV infection would very much depend on the context and setting in which they first learned of it. For example, some women felt that finding out about HPV and its role in cervical cancer in a colposcopy setting would provoke a more intense reaction than learning of it through HPV vaccination. The way in which HPV was explained to them by healthcare practitioners (HCPs) was seen by women to be very important.

### Societal influences

Women considered that the opinions of society about HPV infection would be an important influence on acceptability of, and any stigma associated with, having a HPV infection. They suggested that any potential stigma could be countered in the way in which HPV infection is explained (e.g. emphasising high prevalence).

As regards health issues, most women described deferring to trusted sources such as HCPs or the Department of Health, and thought they would feel this way about HPV also. These women felt that HCPs did not currently provide women with enough information about HPV infection. Even women who did not defer responsibility stated that they would take the opinions of HCPs and the Department of Health regarding HPV and its role in cervical cancer prevention into consideration. The attitude of HCPs was viewed as especially important by women; a positive attitude by a HCP was considered more likely to mitigate women’s concerns about HPV.

### HPV testing

Women tended to perceive that HPV testing was more personally relevant to them than HPV infection. Early in the discussions most women felt that they would want to have a HPV test in addition to a usual cytology test; this resulted from a desire to “take care” of their bodies and to know if they had a HPV infection. However over the course of each discussion, issues such as the prevalence of HPV and a lack of treatment caused most of these women to become less certain about being tested; by the end of each discussion, most considered that undergoing an HPV test would simply cause unnecessary worry. Strong feelings of reliance on existing cervical screening cytology was found as women discussed HPV testing. If testing was to be done, women considered that it would be most acceptable as part of triage for low-grade abnormal cytology tests, since women who tested positive would then undergo some management/follow-up. Women who declared themselves as proactive with regard to preventative healthcare were more likely to be in favour of having HPV tests, and less likely to change their mind about HPV testing during the course of discussion.

Three primary themes emerged in relation to HPV testing: *knowledge*, *logistics*, and *psychological effect* (Additional file 3).

#### Knowledge

Almost all women, even those who were aware of HPV infection and vaccination, lacked any knowledge of HPV testing. Only one woman stated she was aware of HPV testing, having learned of it in relation to having treatment for abnormal cytology.

On learning about the introduction of HPV testing in other countries, some women questioned whether it was more reliable than cytology. However, women were confused about what HPV tests involved and whether HPV testing and cytology tested for the same thing: specifically women questioned how someone could have a positive result for one test, and not the other. Some women thought HPV testing should be offered as a preventative measure to limit HPV transmission.

#### Logistics

Women thought that, if a HPV test was to be conducted, it should, for convenience, be carried out at the same time as a cytological test, and by the same HCP. They were keen for international guidelines and methods of best practice to be followed. Issues such as cost of testing and possible physical discomfort were also raised.

#### Psychological effect

Women spoke about fears of testing HPV positive, due to the possible implications for their health and relationships and fear of the unknown. Women discussed extensively possible feelings of anger and blame within relationships if a woman tested positive. They expressed a desire for men to be tested; and commonly described feelings of anger, or anticipated feeling angry, about how HPV was contracted. They also expressed worries and potential embarrassment about the difficulty of talking to a partner about being HPV tested and/or revealing HPV status due to the sexually transmittable nature of HPV.

Women described feeling “powerless” at the lack of treatment of HPV; this then made them call into question the purpose and value of testing especially when cytology tests were already available.

Women spoke about the worry that could result from waiting for HPV test results. Some women considered that adequate explanation of results would be of paramount important in order to minimise negative psychological effects associated with testing positive. In contrast, others believed that a positive HPV test would be an encouragement to attend for further screening/treatment and receiving a negative HPV test would be reassuring.

## Discussion

### Women’s trust in cytology

Incorporating HPV testing into cervical cancer screening, could change women’s perceptions of cervical cancer and influence sexual attitudes and behaviours in the population. This, in turn, may affect screening participation and its psychological impact. Most leaders in the area anticipate HPV primary screening will be implemented within a few years [15-17]. One of the main findings of this study, however, was that women were concerned with the lack of treatment for HPV infection and showed a preference for existing cytology. This suggests that changing screening from cytology-based to HPV-based may face significant obstacles.

While a national cervical cancer screening programme was not in place in Ireland at the time of this study, many women had had cytology tests, often on an opportune basis such as post-childbirth. This seems to have generated strong feeling of dependence and reliance on cytology. Studies in other settings have also found that women trust cytology and are reluctant to replace it. In an American focus group study [18] nearly all participants were firmly set against reducing the frequency of cytology tests. An Australian study [19] found that 85% of women wanted concurrent cytology and HPV testing. Cytology screening rates are falling in a number of countries [20] and further changes to prevention protocols (such as a move to primary HPV testing) may negatively impact these.

### Role of HCPs and government

The role of HCPs in influencing their patients’ health screening behaviours is well documented [21,22]. This study extended these observations into the arena of HPV. The majority of women deferred responsibility for health prevention to their HCP and governmental health departments, suggesting that women’s attitudes and responses to changes to cervical cancer screening, including perhaps the introduction of primary HPV testing, will be strongly influenced by their relationship with their HCP. Thus any changes to screening will need to be lead and encourage by the government and HCPs. It is of some concern, therefore, that primary care practitioners (who, in Ireland, conduct the majority of cervical screening tests) have significant gaps in their knowledge, and feel considerable uncertainty, about HPV infection, testing and vaccination [23-25].

### Knowledge of HPV infection and HPV testing

In common with studies elsewhere [26-35], in this study women had many unanswered questions about HPV infection and its association with cervical cancer. Specifically there was confusion over source, treatment and re-infection, similar to UK findings [36]. One of the most notable findings was that women’s opinions about HPV testing altered during the discussion: initial support for comprehensive testing transformed into favouring much more limited or reactive HPV testing. Moreover, women made little connection between having a positive HPV test result and being at risk of cervical cancer. The implication of these findings is that it may be difficult for women (with their current levels of knowledge about HPV) to appreciate why prevention strategies are changing and to make informed choices about HPV testing within screening.

### Psychological effects of HPV testing

If HPV testing was unacceptable to the general population, or caused high levels of distress or anxiety among those tested, this would have serious implications for the possible use of HPV testing in routine cervical cancer screening. In this study, the lack of treatment for those who test HPV positive was a major concern for women, who consequently expressed greater confidence in cytology as they knew treatment options are available for those with abnormal results. This suggests that screening programmes may face significant challenges around informing women that they are infected with a high risk “cancer virus” but not offering explicit treatment. This has important implications for education and information initiatives around new screening protocols.

Other studies have suggested that HPV testing may be a sensitive and complex issue for women, confounded by the psychosocial stigmas and distress associated with contracting a STD and its link to cervical cancer [37-39]. Similarly, in this study women frequently described possible feelings of anxiety, fear, stigmatisation and concern about their sexual relationships. However our study also found that the impact of possibly testing positive varied; for some women understanding that HPV is a common infection and can potentially clear up on its own appeared to reduce potential for stigma and embarrassment. The high prevalence also led some women to feel secure about testing positive and helped them disassociate HPV from other STDs. This suggests, for HCPs and screening programmes, that emphasizing how common and “normal” HPV infection is may help minimise adverse psychological effects of HPV testing. Our findings also suggest other ways in which messages about HPV may be best framed. Specifically, moving the focus towards cervical cancer and away from HPV itself may be more acceptable to women. Clearly the provision of adequate and appropriate information for women about HPV will be vital. This, however, is unlikely to be a trivial undertaking since, as we and others have shown, women are likely to vary in their need and desire for information [40]. Johnson et al. [39] found that associations between HPV status and anxiety may be explained by factors other than learning of test results and may vary by ethnicity and lifestyle factors, highlighting the need for tailored information.

### Strengths & limitations

The major advantage of qualitative focus groups is that, as well as gathering participants’ views, the interactions between participants may reveal additional issues. An example of this was the change in women’s opinions about HPV testing as discussions progressed; to our knowledge, this is the first study to identify the evolving nature of women’s responses to HPV. A major strength of the study is that it was carried out soon after the introduction of the HPV vaccination programme and, therefore, is likely to have captured any impact that this had on awareness of HPV amongst the population. Moreover, while these focus groups were conducted some time ago, their messages and implications remain timely; only within the past year have several screening programmes started to introduce HPV testing [2-6].

In recruiting to the focus groups we aimed for maximum diversity of women’s opinions and experiences (e.g. 10% of women never had a smear before, similar to the national population [6]). This diversity was evident both in their characteristics (which reflected the national socio-demographic [41] and % public patients [42]) and the views they expressed. None of the women in the focus groups worked directly in health related areas; however, it is possible that some women volunteering to take part because they had particular interest in the topic (i.e. cervical cancer screening). Another limitation is that, as in other qualitative studies, the relative weight or importance of themes and subthemes is not always clear.

## Conclusions

Despite the changing landscape of cervical cancer prevention, women remain strongly attached to cytology testing. They have concerns with the lack of treatment for HPV infection and this impacted on their preferences on how HPV testing might be accommodated, in an acceptable way, within screening programmes. HCPs will play a crucial role in securing women’s support and compliance with HPV testing. Tailored, appropriate and timely information regarding HPV will be needed to minimise adverse psychological effects and ensure that future cervical cancer prevention strategies continue to be effective.

## Abbreviations

CERVIVA: Irish cervical screening research consortium; GMS: General medical services (part of the Irish primary care reimbursement services); HPV: Human papilloma virus; HCP: Health care practitioner; STD: Sexually transmitted disease.

## Competing interests

No competing interests are held by the authors.

This work was supported by the Health Research Board in Ireland to the Irish Cervical Screening Research Consortium (Cerviva) [HS-05-09].

## Authors’ contributions

JMR carried out the focus groups, coordination of the study and drafted the manuscripts. LS designed the study. CM and JOL participated in the design of the study and read and approved the final manuscript. All authors read and approved the final manuscript.

## Pre-publication history

The pre-publication history for this paper can be accessed here:

http://www.biomedcentral.com/1472-6874/14/64/prepub

## Supplementary Material

Additional file 1 **Appendix: Written information provided to Focus Groups regarding HPV.** From the European Cervical Cancer Association booklet range [http://www.ecca.info/ga/ecca-publications/brochures.html] and used with permission of the ECCA. Click here for file

Additional file 2 Table of socio-demographics of focus group participants. Click here for file

Additional file 3 Table of primary themes, subthemes and illustrative quotes for HPV infection and HPV testing. Click here for file
