# Supplementary material for: “If you can’t treat HPV, why test for it?” Women’s attitudes to the changing face of cervical cancer prevention: a focus group study
Source: BMC Womens Health. 2014 May 6;14:64. doi: 10.1186/1472-6874-14-64 (PMC4135323; doi:10.1186/1472-6874-14-64)
Supplement: Additional file 1 — Appendix: Written information provided to Focus Groups regarding HPV. From the European Cervical Cancer Association booklet range [http://www.ecca.info/ga/ecca-publications/brochures.html] and used with permission of the ECCA. [file 1472-6874-14-64-S1.docx]

**Additional File 1**

**Appendix: Written information provided to Focus Groups regarding HPV**

From the European Cervical Cancer Association booklet range

[www.ecca.info/ga/ecca-publications/brochures.html] and used with permission of the ECCA.

| The following information is taken from the ECCA booklet you will be given in an information pack to take away at the end of the focus group.  **Human Papilloma Virus (HPV) and Cervical Cancer**  **www.ecca.info/ga/ecca-publications/brochures.html** |
| --- |

**What is Human Papilloma Virus (HPV)?**

There are more than 100 types of HPV. About 40 of these can infect the genital area of both men and women, and are called genital HPVs. Some of these cause genital warts and others, about 15 types, may cause abnormal cervical cells that can eventually progress to cervical cancer.

HPV is so common that most adults are likely to have had it at some time in their lives. Fortunately for the majority of people, their immune system will fight-off the HPV and clear it in 6 to 24 months. While a woman has HPV, it can produce the abnormal cervical cells found on her cervical smear, but these also usually disappear once the virus has been cleared.

The problem occurs when some women do not clear their HPV. In these cases, the abnormal cervical cells may progress and develop into cervical cancer if they are not found first and removed. Abnormal cervical cells are very easy to treat, but they have no outward signs or symptoms and the only way they can be found is by cervical screening.

HPV types 6 & 11 cause the majority of cases of genital warts and also a number of mildly abnormal Pap tests. However, the HPV types that cause genital warts are not the same types that can lead to cervical cancer. Therefore, women with genital warts do not have an increased risk of developing cervical cancer and they do not need to be screened more often.

**How is HPV transmitted?**

Genital HPV is usually spread by sexual contact and it is easily transmitted from one person to another by any [genital] skin-to-skin contact, not just sexual intercourse. Because HPV is both common and easily transmitted, most adults will have had it at sometime in their lives.

Although HPV is usually cleared in 6 to 24 months, it can sometimes remain for many years, all the time without any signs or symptoms. Because of this, it is often very difficult to know when or where it came from.

**Can HPV infection be prevented?**

Although condoms are very effective in preventing other sexually transmitted infections, they are not as effective in preventing HPV, probably because HPV can be found on skin not covered by the condom.

Vaccination can prevent infection with certain types of HPV. Studies have shown that the currently available vaccines are very effective in preventing infection by HPV 16 & 18 and may also provide protection against a few other types. However, they will not protect against all types of HPV.

**What is HPV testing?**

HPV testing is used to find out if you have HPV and it can be done on a sample of cervical cells taken in the same way as a cervical smear. Although there currently are no treatments for the HPV types that can cause cervical cancer, finding out if you have HPV or not can help to define your risk for developing this disease. If you do not have HPV, then your risk is very low and you can safely go back to routine screening. If you do have HPV and your immune system does not fight it off, then a follow-up to find out if you have abnormal cervical cells may be needed so they can be removed to prevent cancer developing.

Three possible uses have been proposed:

• as a test to screen women for cervical cancer,

• as a test for women who have mild abnormalities on their Pap test to help decide if follow-up is needed or not,

• as a test to follow-up women who have been treated for abnormal cervical cells to help decide if further treatments are required or not.

**What is HPV vaccination?**

Vaccination is now available to protect against the 2 most common types of HPV, types 16 & 18, which cause about 2 of every 3 cases of cervical cancer and many abnormal Pap tests. However, it will not protect you against all HPV types and even if you have been vaccinated, you should still be screened.

Also, the current vaccines have been designed to prevent HPV infection occurring in the first place. Therefore, it is thought that they will provide the greatest protection if given before the exposure to HPV has occurred, for example, to pre-adolescents before the start of sexual activities. However, infection with the HPV types included in the vaccines can occur at any time and therefore adolescents and adults may also benefit from vaccination even if they have already started sexual activities.
