# Supplementary material for: “If you can’t treat HPV, why test for it?” Women’s attitudes to the changing face of cervical cancer prevention: a focus group study
Source: BMC Womens Health. 2014 May 6;14:64. doi: 10.1186/1472-6874-14-64 (PMC4135323; doi:10.1186/1472-6874-14-64)
Supplement: Additional file 2 — Table of socio-demographics of focus group participants. [file 1472-6874-14-64-S2.docx]

**Additional File 2**

**Socio-demographics of focus group participants**

| **Age of female participants:** | | | | | | | |
| --- | --- | --- | --- | --- | --- | --- | --- |
| **Age range years:** | **17-20** | **21 – 30** | **31-40** | **41-50** | **51-60** | **61-70** | **Total** |
| n (%) | 3 (5.1) | 10 (16.9) | 17 (28.8) | 15 (25.4) | 9 (15.3) | 5 (8.5) | 59 (100) |
| **Relationship Status:** | | | | | | | |
| Single | 3 (5.1) | 1 (1.7) | 3 (5.1) | 1 (1.7) | 1 (1.7) | 0 (0) | 9 (15.3) |
| Married | 0 (0) | 2 (3.4) | 11 (18.6) | 10 (16.9) | 5 (8.5) | 5 (8.5) | 33 (55.9) |
| Cohabiting | 0 (0) | 2 (3.4) | 2 (3.4) | 1 (1.7) | 0 (0) | 0 (0) | 5 (8.5) |
| Divorced/separated | 0 (0) | 1 (1.7) | 1 (1.7) | 3 (5.1) | 3 (5.1) | 0 (0) | 8 (13.6) |
| Widowed | 0 (0) | 0 (0) | 0 (0) | 0 (0) | 0 (0) | 0 (0) | 0 (0) |
| Relationship  (not cohabiting) | 0 (0) | 4 (6.8) | 0 (0) | 0 (0) | 0 (0) | 0 (0) | 4 (6.8) |
| **Education:** | | | | | | | |
| No formal education | 0 (0) | 0 (0) | 0 (0) | 0 (0) | 0 (0) | 0 (0) | 0 (0) |
| Primary education | 0 (0) | 0 (0) | 1 (1.7) | 0 (0) | 4 (6.8) | 1 (1.7) | 6 (10.2) |
| Second level lower | 0 (0) | 1 (1.7) | 2 (3.4) | 2 (3.4) | 1 (1.7) | 0 (0) | 6 (10.2) |
| Second level upper | 3 (5.1) | 2 (3.4) | 4 (6.8) | 4 (6.8) | 1 (1.7) | 0 (0) | 14 (23.7) |
| Third level non-degree | 0 (0) | 5 (8.5) | 5 (8.5) | 6 (10.2) | 1 (1.7) | 3 (5.1) | 20 (33.9) |
| Third level degree | 0 (0) | 2 (3.4) | 5 (8.5) | 3 (5.1) | 2 (3.4) | 1 (1.7) | 13 (22.0) |
| **Cervical screening attendance:** | | | | | | | |
| Never attended | 3 (5.1) | 1(1.7) | 0 (0) | 0 (0) | 1 (1.7) | 1 (1.7) | 6 (10.2) |
| At least attended once | 0 (0) | 9 (15.3) | 17 (28.8) | 15 (25.4) | 8 (13.6) | 4 (6.8) | 53 (89.8) |
| **Private or Public patient:** | | | | | | | |
| Public | 2 (3.4) | 5 (8.5) | 4 (6.8) | 3 (5.1) | 3 (5.1) | 1 (1.7) | 18 (30.5) |
| Private | 1 (1.7) | 5 (8.5) | 13 (22.0) | 12 (20.3) | 6 (10.2) | 4 (6.8) | 41 (69.5) |
