# Supplementary material for: “If you can’t treat HPV, why test for it?” Women’s attitudes to the changing face of cervical cancer prevention: a focus group study
Source: BMC Womens Health. 2014 May 6;14:64. doi: 10.1186/1472-6874-14-64 (PMC4135323; doi:10.1186/1472-6874-14-64)
Supplement: Additional file 3 — Table of primary themes, subthemes and illustrative quotes for HPV infection and HPV testing. [file 1472-6874-14-64-S3.docx]

**Additional File 3 Primary themes, subthemes and illustrative quotes for HPV infection and HPV testing.**

| **Primary themes** | **Subthemes** | **Sample quotes** |
| --- | --- | --- |
| **HPV Infection** |  |  |
| Knowledge | Lack of information  Appropriate information  Link to cervical cancer | DE1 All I can think of about is GP’s [general practitioners] and the doctors and everybody in general in the medical profession actually don’t tell you anything.  DE1 We’re just not being told like the …medical secret about everything  MY2 You don’t want to know the information until it hits your own door.  PB3 Maybe it’s the whole I mean I don’t know how you see this is but maybe if the whole Sexually Transmitted Disease thing was removed slightly from the whole concept of cervical cancer. You know it’s a cancer, it can kill you, it can kill women and younger women are dying of cervical cancer.  PB3 I think the link between maybe HPV and cervical cancer has to be explained better as well because it looks like they are two separate things and they are not. |
| Emotional response | Shock  Anger | LC1 I think to even describe it on the internet as a, as a, did I read there it’s a sort of a sexual disease, I certainly would have never seen it as sexual disease, I would have seen it alright it had a virus but I don’t see myself as having a disease, I got kind of a shock when I saw that because I would be thinking of sexual diseases like.  LC3 I think some women would get very angry if they thought well I’ve only been with him, you know and he’s given this to me now, you know I think that is something that might come up for some people. |
| Societal influences | Stigma  Defer responsibility to trusted sources | PB5 I mean you could have someone who’s never had sex with anybody but her first partner and she might still get the virus I don’t think it should be labelled as a Sexually Transmitted Disease it may be just a virus that happens to be transferable from one body to another body or contact with one body with another body but it doesn’t mean it is something that should be regarded the same as Syphilis or Gonorrhoea or any of those things, I wouldn’t like it to have, to be thought of in the same way.  BM04 For things like this I would probably look up on the internet in work or whatever. But for my sister or my mam [mother] like…they would just go to the doctor and ask the GP[general practitioner]. |
| **HPV Testing** |  |  |
| Knowledge | Preference for HPV testing in addition to smear testing  Preference for smear tests  Testing the same thing | PB1 I think it should be part of the smear test I think I should have been tested for this if it’s anything that can occur because I’d rather have the information, I’d rather, even if it is they’re saying well this is present in the whole population and it’s probably going to clear up but we’re going to monitor it and this is what you have and you’re good to go, oh you’re fine yea, I’d rather know.  NZ8 I mean if the majority of adults are likely to have had it at some time in their lives……And quite happy living in ignorance but as long as your smears are normal, that’s all that would bother me.  NZ2 I think I wouldn’t even want HPV testing actually. I think I’d just go for the smears.  LC6 Well yea because it’s the same, it’s the same process that, like, a smear test, so I think if you just go for a smear test [that would be best]. |
| Logistics | Side-by –side testing  Deferred responsibility | T05 Well if it’s going to let you know whether you have a high risk or a low risk of cervical cancer in the future. I think it definitely should be done side by side with the screening. I mean just as you are there you should have two tests as the one.  PB6 Are there other countries, are we lagging behind other countries who test for this automatically or are leading the field?  PB3 I don’t see the point to it [HPV testing] to be honest, I mean maybe as a test women like number two who have mild abnormalities in the [smear test], I think doctors as well, or clinicians have to decide you know what is clinically right, what does, what are international guidelines on this you know. |
| Psychological effect | Fear  Lack of treatment  Worry  Anger/blame | PB3 I think it would be kind of a, maybe a waste of resources and scary for everybody to be tested for HPV if we all have it anyway and we can’t do anything about it.  FA1 Just the fear of something, just to see that it will show maybe something bad…like.  PB7 Well yea it [diagnosis as HPV positive] leaves you in a very powerless situation.  NZ8 If there was a treatment that they could give you a pill and get rid of it then I’d go and get it done tomorrow. But if there isn’t what’s the point?  PB7 there’s a huge probability will just clear up of its own accord and you could actually get people very worried about something that their body was just going to cope with and process and get rid of naturally  NZ8 Yea. Is there any advantage in knowing that you’ve got it? That’s what I mean. Is knowledge a good thing there or is it better to let your body get on and fight it? ……Ok you know you’ve got it but you can’t do anything about it. Sort of knowledge that you’d be more worried perhaps knowing that you’ve got it  LC5 One of my friends discovered that she had these abnormal cells and she had to go for treatment and everything and she was absolutely raging with her partner because she knew that he you know he had had a life, he was older than her and he had had a life before she came along, she was a virgin whenever she got together with him and had her two boys with him and she was just so angry and so full of hatred towards him that she immediately blamed him. |
|  |  |  |
